# Blood leukocyte composition and function in periparturient ewes kept on different dietary magnesium supply

**DOI:** 10.1186/s12917-020-02705-9

**Published:** 2020-12-14

**Authors:** Mona H. Ahmed, Mirja R. Wilkens, Bernd Möller, Martin Ganter, Gerhard Breves, Hans-Joachim Schuberth

**Affiliations:** 1 Institute of Physiology and Cell Biology, University of Veterinary Medicine Hannover, Foundation, Bischofsholer Damm 15, D-30173 Hannover, Germany; 2 Institute of Farm Animal Genetics, Friedrich-Loeffler-Institute (FLI), D-31535 Neustadt, Mecklenhorst Germany; 3 Clinic for Swine, Small Ruminants and Forensic Medicine, University of Veterinary Medicine Hannover, Foundation, Bischofsholer Damm 15, D-30173 Hannover, Germany; 4 Institute of Immunology, University of Veterinary Medicine Hannover, Foundation, Bünteweg 2, Building 261, D-30559 Hannover, Germany

**Keywords:** Magnesium, Neutrophil phagocytosis, Monocyte subsets, Lymphocyte proliferation, Vaccination, Transition period, Sheep

## Abstract

**Background:**

Transition period (TP) is characterised by physiological and metabolic changes contributing to immunodysregulation. Since knowledge about this period in sheep is scarce, we analysed changes in selected immune variables during the TP in ewes and whether dietary magnesium (Mg) supplementation could modulate these immune variables. Pregnant ewes (2nd and 3rd lactation) were divided into a control group (CONT, *n* = 9) and a Mg group (MAG, *n* = 10) supplemented with Mg oxide resulting in a daily Mg intake of approximately 0.30 and 0.38% (MAG) of dry matter during ante- (a.p.) and post-partum (p.p.) periods, respectively. Blood samples were collected between days (d) 30 a.p. and d 30 p.p.. Whole blood neutrophil phagocytic activity, monocyte subset (classical cM, intermediate intM, non-classical ncM) composition and the proliferative capacity of lymphocytes were determined flow cytometrically. At d 14 a.p., all ewes were vaccinated against *Mycobacterium avium subsp. paratuberculosis* (MAP).

**Results:**

Both groups showed a sharp increase in the total leukocyte counts (TLC) and neutrophil counts (*P* < 0.0001), at d 1 p.p., while, monocytes and their subpopulations displayed the highest values at d 30 p.p. (*P* ≤ 0.05). At d 1 p.p. the neutrophil phagocytic activity was higher (*P* < 0.05) in MAG ewes. Throughout the TP, the proliferative response of CD4+ cells was significantly higher in the MAG group (*P* < 0.05). Ewes in both groups responded with an increase in the TLC, neutrophil numbers (*P* ≤ 0.05) and ncM (*P* < 0.001) 24 h post vaccination, whereas monocytes and cM dropped in numbers (*P* ≤ 0.05). Numbers of intM only increased in MAG ewes (*P* < 0.05), whereas lymphocyte numbers decreased (*P* < 0.01). Mg supplementation did not affect the significant increase in MAP-specific antibodies at d 7 and 21 post vaccination. Total Mg and Ca serum levels did not show any differences between the two groups.

**Conclusion:**

Whereas TP-associated fluctuations in blood leukocyte numbers are not influenced by Mg supplementation, neutrophil phagocytic activity, the proliferative capacity of CD4+ cells and the cellular response within 24 h after a vaccination are subject to modulation.

**Supplementary Information:**

The online version contains supplementary material available at 10.1186/s12917-020-02705-9.

## Background

Dramatic changes are observed in the metabolic and endocrine profiles as well as the immune responses in farm animals during the transition period (TP) [1, 2]. As in cows, the TP in ewes covers the interval from 3 weeks before parturition to 3 weeks after parturition [3]. Similar to other farm animals, pregnant ewes undergo metabolic, hormonal and immunological changes to accommodate the fetus’s needs mainly during late pregnancy until early lactation [4]. Immune dysregulation occurs commonly during the TP, this being due to hormonal fluctuations (e.g. progesterone, cortisol, oxidative stress, negative energy balance and mineral imbalances [5, 6]. Changes in immune mechanisms during the TP were reported mainly in cows and to some extent in ewes, such as a reduction in phagocytic activity of neutrophils and macrophages, alteration in the composition of circulating monocyte subsets, production of cytokines, complement activation, proliferation of lymphocytes, and the production of antibodies [7–9]. Accordingly, cows show greater susceptibility to a wide range of diseases such as mastitis and retained placenta, while ewes become more vulnerable to gastrointestinal nematodes peri-parturient rise (PPR) in faecal egg counts [10]. During late pregnancy and early lactation ewes experience a rise in faecal worm egg counts [10–12] which has been linked to the fact that ewes exhibit a range of impaired manifestations of resistance including the ability to resist establishment of newly acquired larvae [13], the ability to suppress worm fecundity [11, 13, 14] and in particular, the ability to expel adult worms [11]. A number of studies have reported that the magnitude of the PPR can be regulated by the dietary supply of metabolisable protein [15, 16] and the host genotype [17].

At cellular level, magnesium (Mg) is required for more than 600 metabolic reactions as a coenzyme or substrate [18]. Mg is an essential component of DNA replication and repair, RNA transcription, amino acid synthesis, and protein formation. In addition, Mg is an important regulator of many enzymes involved in glycolysis, such as adenine nucleotides [18]. It is well known that, Mg availability is of major importance for glucose metabolism and insulin function [19], as it has been observed in diabetic humans and hypomagnesaemic sheep [20–22]. Activation of cellular proliferation is initiated by growth factors that increase glucose uptake and protein synthesis [23], since Mg is a key factor in both processes and in the activation of mammalian target of rapamycin (mTOR) complex [24]. Thus, Mg is undoubtedly involved, in cell signalling and proliferation, which confirms its immunomodulatory potential [25–27].

Although the underlying mechanisms remain unknown, plasma Mg is kept within the range of 0.9–1.2 mmol/L, provided that the influx via absorption from the forestomachs region into the extracellular space exceeds the efflux into the soft tissue and bones for foetal growth during pregnancy, milk production, and intestinal and urinary endogenous secretion [28]. Mobilisation of Mg from bone is unlikely because the ratio Ca:Mg is 42:1 which would disrupt Ca homeostasis [29]. Therefore, absorption from the forestomach region is probably the key factor determining plasma Mg levels, which can only be kept constant when the daily requirement is adequately balanced by ruminal absorption [28]. In ewes, Mg requirement is increased significantly during late pregnancy and early lactation. Therefore, subclinical hypomagnesaemia could occur during the TP in ewes [30].

This knowledge led us to hypothesise that Mg supplementation during the TP of ewes modulates mineral homeostasis, improves glucose metabolism and insulin function as well as distinct immunological variables such as blood leukocyte composition, neutrophil phagocytosis, lymphocyte proliferative capacity, and the response to vaccination.

## Results

### Blood leukocytes

**Fig. 1:**
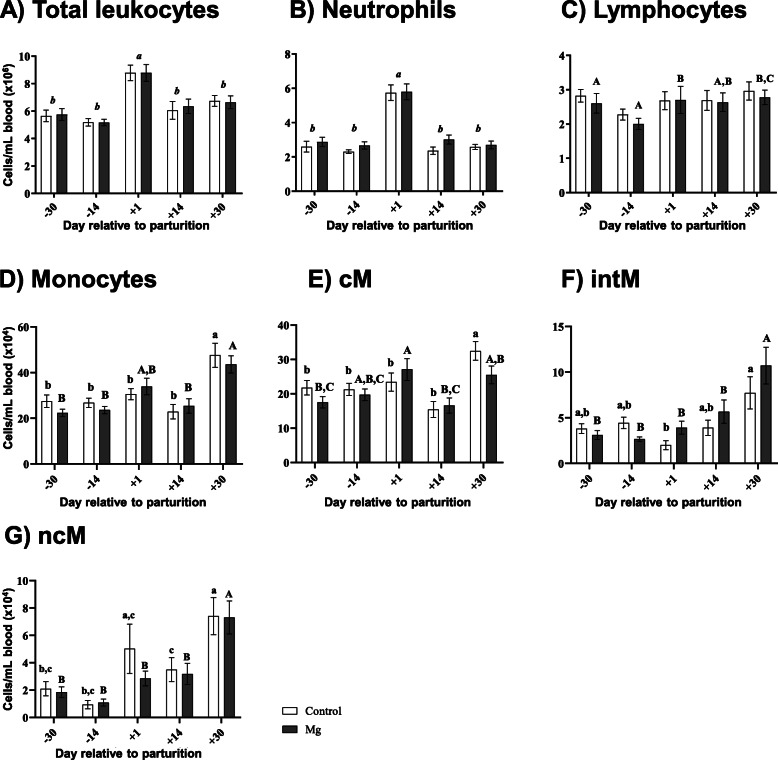
Total leukocyte, neutrophil, lymphocyte, monocyte and monocyte subsets counts in ewes during the transition period and the effect of magnesium supplementation. The two-way ANOVA test and Sidak multiple comparisons test were used for comparison between the different time points and groups. Significant time-dependent differences (*P* ≤ 0.05) are indicated by different small letters for the control group, capital letters for the Mg group and small italic letters for both groups. Mean ± SEM, (control group *n* = 9, Mg group *n* = 10)

At d 1 p.p., a significant increase was observed in TLC and neutrophil counts (*P* < 0.0001) (Fig. 1a, b). This was similar in the control and Mg group. Lymphocyte numbers were significantly lower at d 14 a.p. in the Mg group (*P* < 0.05) (Fig. 1c). Total numbers of monocytes and monocyte subpopulations (cM, intM and ncM) displayed the highest values at d 30 p.p. (*P* ≤ 0.05) (Fig. 1d, e, f, g).

### Neutrophil phagocytic activity

**Fig. 2:**
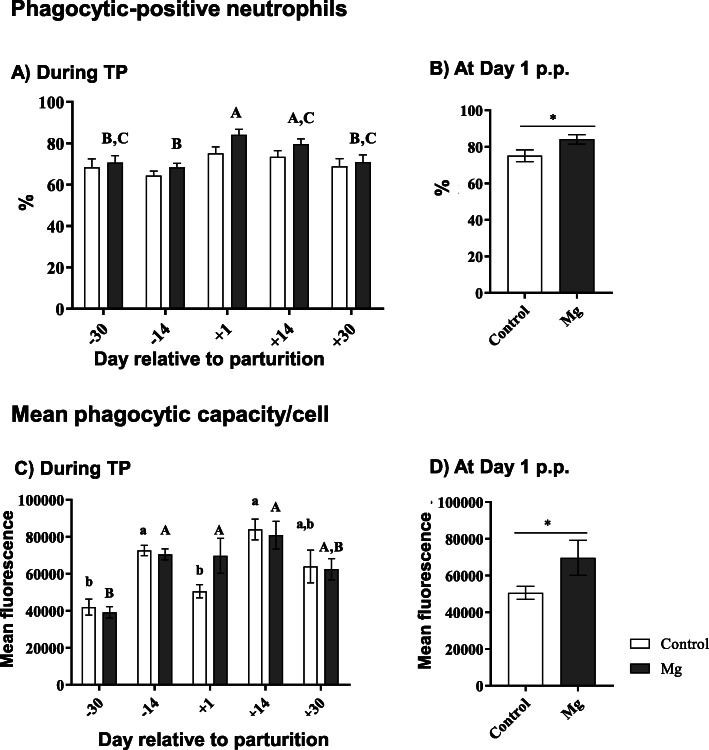
Neutrophil phagocytic activity in vitro (% phagocytic positive neutrophil and mean phagocytic capacity/cell) in ewes during the transition period and the effect of magnesium supplementation. Unpaired t-test, two-way ANOVA test and Sidak multiple comparisons test were used for comparisons between time points and groups. Significant time-dependent differences (*P* ≤ 0.05) are indicated by different small letters for the control group and capital letters for the Mg group. Mean ± SEM, (control group *n* = 9, Mg group *n* = 10). Significant differences between the two groups are indicated as * (*P* < 0.05)

The fraction of phagocytosis-positive neutrophils was lower during the ante-partum period (d 30 a.p. and d 14 a.p.) compared to the post-partum period (d 1 p.p., d 14 p.p. and d 30 p.p.) (Fig. 2a) in both groups. At d 1 p.p. a higher percentage of phagocytosis-positive neutrophils was observed in the Mg group (*P* < 0.05) (Fig. 2b). The mean phagocytic capacity per cell changed more clearly through the TP (Fig. 2c) with lowest values in the ante-partum period in both groups (*P* < 0.05–0.0001). At d 1 p.p. ewes supplemented with Mg displayed significantly higher neutrophil phagocytic capacity (*P* < 0.05) compared to the control ewes (Fig. 2d).

### Lymphocyte proliferation

**Fig. 3:**
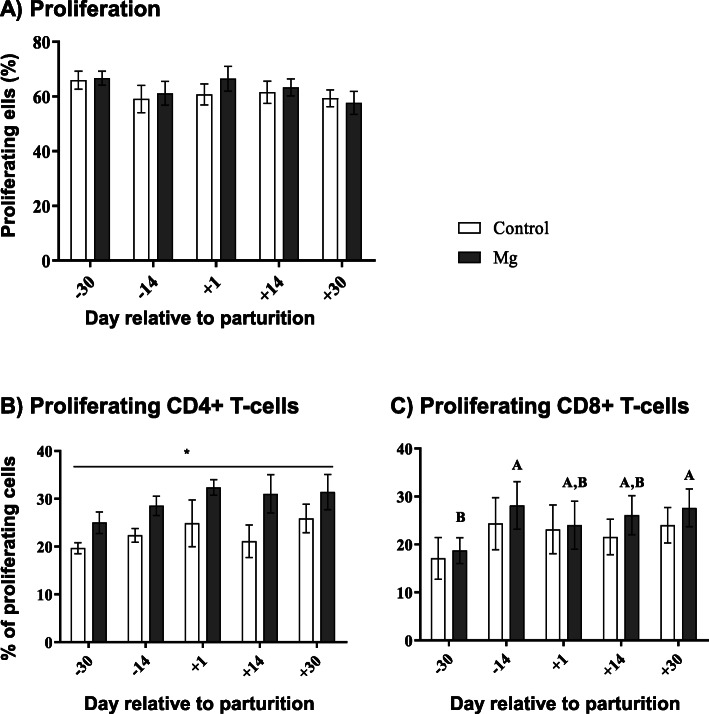
T lymphocytes proliferative capacity as well as CD4+ and CD8+ T cells response in vitro in ewes during the transition period and the effect of magnesium supplementation. Two-way ANOVA test and Sidak multiple comparisons test were used for comparisons between time points and groups. Significant time-dependent differences (*P* ≤ 0.05) are indicated by capital letters in the Mg group. Mean ± SEM, (control group *n* = 9, Mg group *n* = 10). Significant differences between the two groups are indicated as * (*P* < 0.05)

Neither the time nor the Mg supplementation had significant effects on the proliferative capacity of all lymphocytes throughout the TP (Fig. 3a). However, Mg supplemented ewes showed a higher proliferative capacity of CD4+ T lymphocytes (*P* < 0.05) compared to the control ewes (Fig. 3b). In addition, only in the Mg group there was a significant time dependent effect on the proliferative capacity of CD8+ T-cells (*P* < 0.01, Fig. 3c).

### MAP vaccination response

**Fig. 4:**
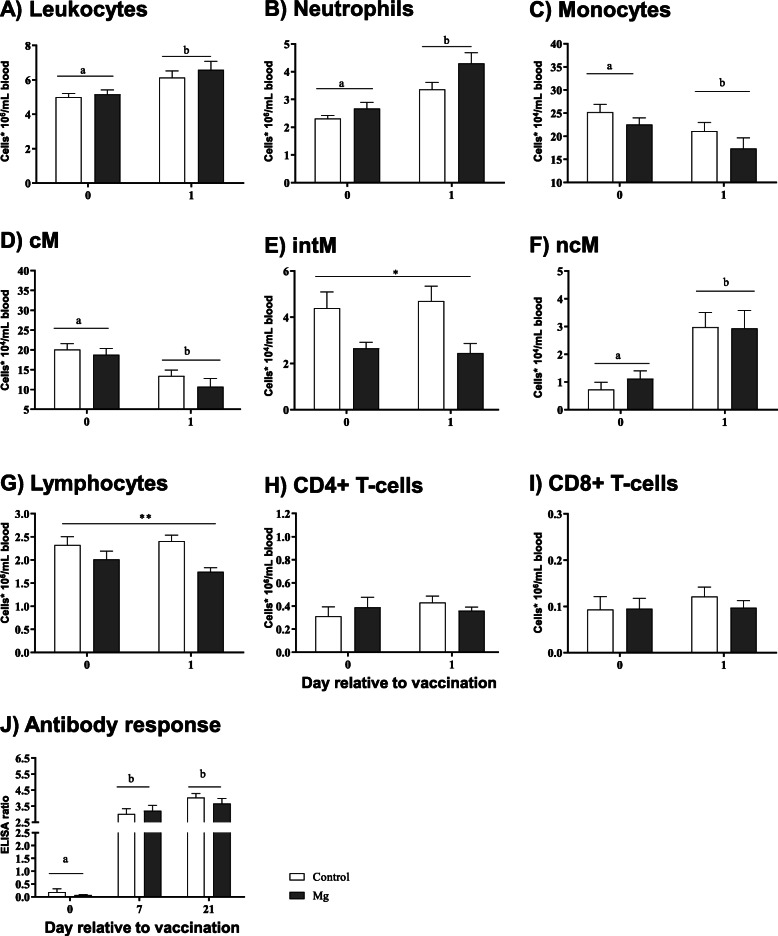
*Mycobacterium avium paratuberculosis* MAP vaccination induced cellular and humoral response in ewes during the transition period. Ewes were vaccinated at d 14 a.p, blood samples were collected before the vaccination (0) and 24 h following the vaccination (1) to investigate the cellular compositions (**a**, **b**, **c**, **d**, **e**, **f**, **g**, **h** and **i**), whereas serum samples were collected at d 7 and d 21 postvaccination to assess the MAP Abs response (**j**). Two-way ANOVA test and Sidak multiple comparisons test were used to detect differences between control (*n* = 9) and Mg (*n* = 10) groups as well as between time points. Significant time-dependent differences (*P* ≤ 0.05) are indicated by different letters. Significant differences between the two groups are indicated as* (*P* < 0.05) and ** (*P* < 0.01). Mean ± SEM

Transient enrichment in TLC and neutrophil numbers was observed in the peripheral blood within 24 h following the vaccination in both groups (*P* < 0.001 and *P* < 0.0001, respectively) (Fig. 4a, b). However, a depletion was observed in monocyte (*P* < 0.001) and cM (*P* < 0.0001) numbers (Fig. 4c, d). While ncM showed a significant increase (*P* < 0.0001) in response to vaccination in both groups (Fig. 4f), intM numbers increased significantly (*P* < 0.05) only in the Mg group (Fig. 4e). Interestingly, a significant decrease in lymphocyte numbers was observed within 24 h only in the Mg group (*P* < 0.01) (Fig. 4g). In contrast, CD4+ and CD8+ T cells remained unaltered (Fig. 4h, i).

The single vaccination dose successfully stimulated a significant increase in the antigen-specific antibody (MAP Ab) values (ELISA ratios) at days 7 and 21 post vaccination in both groups (*P* < 0.0001) (Fig. 4j). However, there were no significant differences between the two groups.

### Faecal worm egg counts and eosinophil numbers

**Fig. 5:**
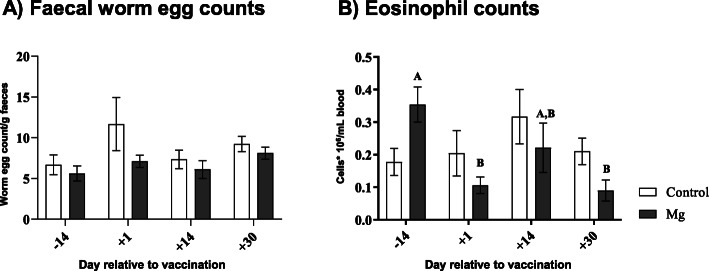
Faecal worm egg counts (**a**) and blood eosinophils numbers (**b**) in ewes during the transition period and the effect of magnesium supplementation. The antilogarithmic values of log-transformed faecal egg counts were compared with two-way ANOVA test and Sidak multiple comparisons test. Mean ± SEM, (control group *n* = 9, Mg group *n* = 10). For the eosinophil numbers, significant time-dependent differences (*P* ≤ 0.05) are indicated by different capital letters in the Mg group

The faecal worm egg counts were obtained as total numbers/g faeces. The highest faecal worm egg count was observed at d 1 p.p. (*P* > 0.05) only in the control ewes, however, huge variations were observed between the animals during this time point. In the control group the values ranged between 26 to 3316 egg/g faeces. In the Mg group the values ranged between 44 to 298 egg/g faeces. Afterwards, constant values were observed in both groups. Nevertheless, neither the time nor Mg supplementation had an effect on the faecal worm egg counts (Fig. 5a).

During the ante-partum period (d 14 a.p.) ewes offered more Mg showed higher eosinophil count compared to the control ewes (Mg supplementation x Time interaction, *P* < 0.001), whereas the control ewes showed the highest numbers during the post-partum period (*P* < 0.05). At d 1 p.p. and d 30 p.p. a decline in the eosinophils numbers was observed in both groups (Fig. 5b). Additionally, no correlations were observed between the faecal egg counts and blood eosinophil numbers during the ante partum and the post partum periods in both groups.

### Serum mineral and cortisol levels

Data on mineral and cortisol levels were provided in detail elsewhere (Ahmed et al. Res. Vet. Sci., in revision). Briefly, in both groups, neither the time nor the treatment had a significant effect on the total Mg serum levels. Control ewes showed a significant decrease (*P* < 0.05) in the total Ca serum levels at d 1 p.p. compared to d 30 a.p. In addition, at d 14 a.p. Ca/Mg ratios were lower compared to d 30 a.p. and d 14 p.p. (*P* < 0.05). Ca and Ca/Mg values did not show differences throughout the TP in Mg ewes. Neither the time nor the Mg supplementation had significant effects on the serum cortisol levels throughout the TP. The obtained values showed huge variations among the animals. In both groups, the lowest values were reported at d 14 a.p., while the highest values were recorded at d 30 p.p. in the control group, and at d 14 p.p. in the Mg group.

### Lambs born per group

Forty lambs were born in total (control group: 19 lambs; Mg group: 21 lambs). Two ewes of the control group had single lambs, four ewes had twins and three ewes had triples. One ewe in the Mg group had a single lamb, seven ewes had twins, and two ewes had triples.

## Discussion

Substantial changes are observed in the metabolic and endocrine profile as well as in the immune response in farm animals during the TP [1, 2] concurrent with a high incidence of infectious diseases. Accordingly, the current study aimed to analyse changes in selected immune parameters in ewes during the TP and whether dietary Mg supplementation has an impact on these parameters. Based on the values of mineral concentrations and blood cell numbers reported in the present study the ewes were in normal physiological conditions [31].

Regarding the fluctuations in the circulating immune cells during the TP, blood cell numbers obtained in the present study behaved differently throughout the whole period. Total leukocytes and neutrophils reached a maximum at d 1 p.p. in both groups, similar results previously being observed in ewes [10, 32] and cows [7, 33]. The reason for neutrophilia around parturition is commonly due to high cortisol levels at this time, which contributes to downregulation of surface adhesion molecules expression [34] and possibly to up regulation of anti-adhesion molecules [35, 36], in addition to an enhanced release of cells from the bone marrow [37]. However, we did not observe a cortisol peak at parturition as has been previously reported in cows [37, 38]. Moreover, cortisol values obtained in the present study throughout the TP were substantially higher than those previously reported for sheep [39, 40], which might have been due to capturing and fixation of the animals for blood sampling.

Ovine blood monocyte numbers peaked later at d 30 p.p. This kind of fluctuation in monocyte cell numbers during the TP was true for all identified monocyte subpopulations, which is in contrast to respective findings in cows [7] where these cells peaked together with neutrophils at parturition [7]. This might be reflected by the fact that that ovine monocytes display a different redistribution pattern during the TP compared to bovine counterparts. The obtained results showed high similarity in the classification system for ovine monocyte subsets with human and bovine classical, intermediate and non-classical monocytes [41, 42] and the results are comparable to previous ovine studies [43, 44]. However, to the best of our knowledge this is the first study to investigate the kinetics of ovine blood monocyte subsets during the TP.

Blood lymphocyte numbers showed the lowest values before parturition and then increased at parturition which is in line with previous study in cows [45], whereas stable numbers throughout the TP were reported by Eger et al., [7] and Cui et al., [27].

During late pregnancy and early lactation ewes generally experience a rise in faecal worm egg counts (Periparturient rise (PPR)) [10], which is associated with a temporary decrease in host immunity as reported by reduction in the circulating eosinophil numbers and antibody levels against specific nematodes [10]. In the present study, we weren’t able to detect a classical PPR, maybe due to high variation in faecal egg count among the animals, however, the highest faecal worm egg count was reported at d 1 p.p. in the control ewes, a larger group size might be needed to detect significant differences. In parallel blood samples were collected to determine the blood eosinophil numbers, the highest value was observed at d 14 p.p. in the Mg ewes which point towards an interaction between Mg supplementation and time, whereas the lowest values were observed at d 1 p.p. and d 30 p.p. in the both groups, our findings regarding the decline in eosinophil numbers at parturition are in line with a previous study [10]. A negative association between blood eosinophil numbers and faecal worm egg counts was reported previously in ewes during late pregnancy and parturition as well as early lactation periods [10] which could point towards a link between PPR and broader tissue changes (parturition and lactation) associated with relaxation or redirection of immune responsiveness. Further research is needed to investigate the interaction between Mg supplementation and PPR phenomenon in ewes.

In the present study, the percentage of phagocytosis-positive ovine neutrophils did not change substantially during the TP with lower values during the ante-partum period. However, the individual cell capacity for phagocytosis changed more drastically, with highest values at d 14. p.p.. Since the phagocytosis test system did not discriminate between adhesion of FITC-labelled *S. aureus* and the ingestion of the bacteria, this could either indicate that the higher mean fluorescence values of ovine neutrophils is due to an enhanced uptake of FTIC-labelled *S. aureus* and/or due to an enhanced concentration of phagocytosis/adherence-favouring opsonizing factors (e.g. complement factors) in blood plasma. This change in the phagocytic capacity of ovine neutrophils during the TP is in line with a study in cows [46], while it contrasts to the data demonstrated by Batistel et al. [47], who reported lowest phagocytosis values at d 1 p.p.. In the present study, at d 1 p.p., ewes supplemented with Mg showed both a significantly higher percentage of phagocytosis-positive neutrophils, as well as a higher phagocytic capacity per cell compared to the control ewes. Comparable studies are scarce. Our findings are supported partly by in vitro data obtained with a monocyte-like human lymphoma U937 cell line where extracellular Mg levels correlated positively with an enhanced phagocytosis rate [48]. The enhancement in the phagocytic potential of neutrophils could be due to the enhancement in glucose viability and cellular uptake [19, 49]. Noteworthy, glucose levels showed less fluctuation throughout the TP in Mg supplemented ewes compared to the control group (data not shown). Moreover, ewes offered more Mg showed lower glucose levels compared to the control group at d 1 p.p., which could be linked to higher insulin sensitivity and increased glucose disposal in Mg supplemented ewes [49].

We extended the analysis of Mg supplementation effects on immune functions to the proliferative capacity of lymphocytes. This function appeared to be rather stable throughout the TP when considering the proliferation of all lymphocytes with no apparent influence of Mg supplementation. These findings were in parallel with Mg-supplemented rats whose in vitro ConA-simulated splenocytes proliferated in a comparable way as cells in a control group [50].

From a physiological point of view, the stable pattern of lymphocyte proliferation obtained in the present study was in contrast to the bovine system where the proliferative capacity of lymphocytes was significantly lower around parturition [33] and increased gradually during the postpartum period [38, 51, 52] . In cows, the reduction in proliferative responses around parturition was linked to high cortisol levels during this period [38]. The lack of such cortisol fluctuations in the ewes in our study may partially explain the differences to the bovine system.

The analysis of proliferating T lymphocyte subpopulations revealed some interesting details. In comparison to the control group, the proliferative response of CD4+ T cells in Mg-supplemented ewes was significantly higher throughout the whole TP. In contrast, the effects of Mg supplementation were less prominent on the proliferative capacity of CD8+ T cells, pointing towards a specific role of Mg for T-cell subpopulations. The enhanced CD4+ T cell proliferation could be due to the improvement in glucose viability and cellular uptake [19, 49], as the activation of cellular proliferation is initiated by growth factors that increase glucose uptake and protein synthesis [16]. The role of Mg in lymphocyte signaling pathway activation has been previously reported in human patients diagnosed with a mutation in an Mg transporter gene MAGT1 (novel X-linked human immunodeficiency). This condition is characterised by hypomagnesaemia, CD4+ lymphopenia and defective T-lymphocyte activation [53]. Knockout of another Mg transporter TRPM7 in chicken lymphocyte cell line stopped the lymphocyte proliferation activity in vitro, however, when Mg was added to the culture medium the cells resumed their proliferation [54]. Where Mg plays a role in the activation of T lymphocytes is still a matter of debate. Mg may be involved in T cell receptor activation [53], the glycolysis process [54], or in the activation-induced Ca influx which partially depends on Mg [53].

Apparently, oral Mg supplementation displayed a modulatory role for ovine neutrophil and CD4+ T cell function measured ex vivo. The question whether Mg supplementation also affects complex immune responses depending on the complex interplay of soluble mediators and different cell types in vivo was addressed by analysing the immediate cellular response within 24 h following vaccination and the humoral immune response against *Mycobacterium avium subsp. paratuberculosis* (MAP) at days 7 and 21 post vaccination. These were rather early time points, since the follow up analysis of MAP vaccinations in the field includes the examination of subcutaneous nodules at the inoculation site between 15 to 30 days post vaccination [55], or determination of the IFN- α serum concentration and MAP-specific antibody levels within 1 or 2 months post vaccination [55, 56]. In the present study, the vaccination resulted in a significant increase in MAP-specific antibody levels at d 7 and d 21 postvaccination. This rapid increase in IgG antibodies against MAP can be interpreted as a booster response of ewes, previously infected with MAP [57]. Since there were no significant differences in the antibody response between control and Mg supplemented ewes, this argues against an influence of Mg supplementation on the cascades leading to an activation of existing T and B memory cells.

Effects of vaccination could be observed much earlier (within 24 h): In both animal groups as blood total leukocyte, neutrophil and ncM numbers significantly increased whereas total numbers of blood monocyte and cM dropped post vaccination. The selective influence of Mg supplementation was apparent for blood intM and lymphocyte numbers in Mg supplemented ewes compared to the control ewes.

The observed changes in blood leukocyte subpopulation numbers are most likely due to distal effects of the spectrum of vaccination-induced mediators. Thus, differences between the control and the Mg-supplemented group might point towards an influence of dietary Mg on the regulated release of such factors from vaccine/adjuvant-triggered cells, e.g. dendritic cells as has been reported previously [58, 59]. To the best of our knowledge the influence of Mg on the early innate immune response following vaccination has not been addressed so far.

## Conclusion

Transition period in ewes is associated with fluctuations in blood myeloid cell numbers, which are only partially comparable with those of cows. A significant depression in selected immune cell functions does not occur during the ovine TP. This species-specific circulatory behaviour and function of immune cells during the TP should be considered when ewes are used as model animals. Dietary Mg supplementation does not interfere to a large extent with the circulation behavior of immune cells. In fact, it selectively favours the functional capacity of neutrophils and T-lymphocyte subsets during the TP. The impact of dietary Mg on the composition of circulating immune cells after vaccination against MAP suggests that Mg modulates early vaccine-induced innate immune mechanisms. Whether this holds true for different vaccines needs further analysis.

## Methods

### Animals and feeding regimes

#### Animals

Twenty-three German Blackhead Mutton ewes (entering the 2nd and 3rd lactation, age: 2–3 years, weight: 85–100 kg) were synchronized with Chronogest CR intravaginal sponges (20 mg, flugestone acetate, MSD Santé Animale, Beaucouzé, Cedex, France), and mated naturally with three rams from the same breed. At day 39 post-mating, ewes were transabdominal scanned for pregnancy by using a diagnostic scanner, out of the 23 ewes, nineteen became pregnant.

The 19 clinically healthy pregnant ewes were randomly divided into two groups: control group (*n* = 9) and Mg group (*n* = 10). A sample size calculation was not performed The animals belong to the Federal Research Institute for Animal Health, Institute of Farm Animal Genetics, Friedrich-Loeffler Institute (FLI), Mecklenhorst, Germany. The ewes were housed in the facilities of the Institute. At the end of the experiments the animals were released. An owner consent was not required.

#### Feeding regimen

In brief, the animals were group-fed under surveillance of the personnel. Throughout the experimental period, there were no significant refusal. The ewes received grass silage (3 kg per ewe and day, 32% dry matter, DM) and concentrate (500 g per ewe and day, 89% DM). While the control group was fed a common concentrate available for sheep (Raiffeisen Schafe S2 lose, Agravis Niedersachsen-Süd GmbH, Hannover, Germany) containing 0.29% Mg (as fed), the pellets for the Mg group had been additionally supplemented with magnesium oxide to a final content of 0.51% (as fed). After parturition (48 h post-partum), the amount of concentrate was increased to 1200 g per ewe and day (as fed). Ante-partum, the estimated daily Mg intake was 2.97 g per ewe in the control group (0.21% of DM) and 4.19 g per ewe in the Mg group (0.30% of DM). Postpartum, the estimated daily Mg intake was 4.96 g per ewe in the control group (0.24% of DM) and 7.74 g per ewe in the Mg group (0.38% of DM). Ewes were allowed to adjust to this diet for 2 weeks before starting the experiment (adaptation period). The groups were housed separately with water available ad libitum. No anthelmintic treatment was applied for these animals during the experimental period.

### Blood collection

Blood was obtained by jugular vein puncture into K_2_E (EDTA), sodium heparin, and Clot Activator Tube (CAT) vacutainer tubes (BD Vacutainer systems, Roborough**,** UK) at five time points: d 30 a.p., d 14 a.p., d 1 p.p., d 14 p.p. and d 30 p.p. at 08:00 before the morning feeding. Serum was separated by centrifugation of clotted blood (3000 g, 10 min, 4 °C) and stored in aliquots at − 20 °C for further analysis.

### Serum variables

Total serum calcium levels were measured using a commercial kit (Labor + Technik LT-SYS, Labor + Technik Eberhard Lehmann GmbH, Berlin, Germany) spectrophotometrically (Uvikon XL UV-Visible, Scanning spectrophotometer, Biotek Instruments Inc., Winooski, VT, USA). Total Mg, was determined by using commercial kits (Labor + Technik LT-SYS, Labor + Technik Eberhard Lehmann GmbH) in the diagnostic laboratory of the Clinic for Swine, Small Ruminants and Forensic Medicine, University of Veterinary Medicine Hannover, Foundation, Hannover, Germany. Serum cortisol levels were estimated using ABNOVA® Sheep cortisol ELISA kits (Abnova, Taoyuan, Taiwan) in accordance with the manufacturer’s instructions.

### Vaccination

At d 14 a.p., the ewes in both groups were injected s/c with 1 mL of a commercial vaccine against *Mycobacterium avium paratuberculosis* (MAP), strain 316 F, (Gudair®, CZ Veterinaria, S. A, Pontevedra, Spain). Whole blood samples were collected immediately before the injection (0) and 24 h following the vaccination (1), to assess vaccination-associated changes in the composition of blood leukocytes (neutrophils, lymphocytes, CD4+ and CD8+ cells, monocytes, and monocyte subsets) see below. At d 0, d 7 and d 21 post vaccination serum samples were taken and transferred to the diagnostic laboratory of the Clinic for Swine, Small Ruminants and Forensic Medicine University of Veterinary Medicine Hannover, Foundation, Hannover, Germany, to determine the level of MAP-specific antibodies (MAP Abs) using a commercial diagnostic indirect ELISA (Cattletype® MAP Ab, Indical bioscience, Leipzig GmbH, Germany) in accordance with the manufacturer’s instructions. Results are given in optical densities (OD). Mean values (MV) of the ODs for the negative (NC) and the positive Control (PC) were calculated. The ratio of sample OD to mean OD of the positive control (S/P) was calculated according to the following equation:

$$ \frac{S}{P}= OD\  Sample- MV\  OD\  NC\div MV\  OD\  PC- MN\  OD\  NC $$

As suggested by the manufacturer, an S/P ratio of ≥0.4 was considered positive.

### Total leukocyte counts

Whole heparinised blood (50 μL) was mixed with 450 μL Turk’s solution, and 20 μL were applied to a Neubauer chamber. Leukocytes were counted microscopically (Nikon microscope ECLIPSE 80i). Fractions of neutrophils, lymphocytes and monocytes among blood leukocytes were determined flow cytometrically (Figure S1). For this purpose, 100 μL whole heparinised blood was mixed with 500 μL distilled water (DW) for 3 s, followed by addition of 500 μL double concentrated phosphate buffered saline (2xPBS). After centrifugation (517 x g, 4 min, 8 °C), the supernatant was discharged and the cell pellet was resuspended in PBS. In the case of the remaining erythrocytes, the hypotonic lysis step was repeated until complete erythrolysis. The last cell pellet was suspended in 100 μL PBS (2 μg/mL propidium iodide) and the cell suspension was measured by flow cytometry (BD Accuri C6 flow cytometer®, Becton Dickinson Inc., Holdrege, NE, USA). For each sample 20,000 events were collected. After setting regions to identify propidium iodide-negative (viable) cells (Figure S1-A), identification of singlets among viable leukocytes was performed in a forward scatter area (FSC-A) versus FSC-height density plot A (Figure S1-B). Afterwards, granulocytes (neutrophils), lymphocytes and monocytes were determined among the singlets based on their characteristic forward scatter area (FSC-A) and side scatter area (SSC-A) properties (Figure S1-C). The percentages of the major leukocyte subpopulations (lymphocytes, monocytes and granulocytes) were determined. The obtained percentages were multiplied with the total leukocyte counts to obtain total numbers of these cell types among leukocytes.

### Neutrophil phagocytic activity in whole blood samples

In vitro phagocytosis was performed as previously described [60] with some modifications. Fresh heparinised whole blood (100 μL) was incubated with heat-killed, FITC-labelled *Staphylococcus aureus* (0.5 × 10^9^ bacteria in 400 μL PBS) (Institute of Microbiology, University of Veterinary Medicine, Hannover, Germany). In 1 mL vials; this bacteria suspension was added to 100 μL blood to achieve a bacteria/neutrophil ratio of 50:1. The needed volume of bacteria suspension was calculated based on total numbers of neutrophils/mL blood. Mixtures were incubated for 30 min (37 °C, 5% CO_2_). Blood samples with added PBS (same volume as the bacteria suspension) served as controls. Thereafter, blood/bacteria mixtures were subjected to a hypotonic lysis step by adding 500 μL D. W for 3 s followed by adding 500 μL 2xPBS. The mixture was centrifuged (517 x g, 4 min, 8 °C) and the cell pellet was resuspended in 1 mL PBS (2 μg/ml PI). Neutrophil phagocytic activity was defined flow cytometrically as the percentage of green fluorescent granulocytes (cells were identified based on forward/side scatter characteristics) among viable (PI negative) granulocytes after excluding eosinophils on FITC-fluorescence vs SSC-A density plots (Figure S2-A-D). The mean cellular phagocytic capacity was defined as the geometric mean green fluorescence of phagocytosis-positive (green fluorescing) granulocytes.

### Gradient density separation of mononuclear cells

Separation of mononuclear cells was performed as described previously [41] with some modifications. A total of 20 mL fresh EDTA blood was diluted 1:1 PBS, layered gently on the top of 15 mL lymphocyte separation medium (Density: 1.077 g/mL, Capricorn Scientific GmbH, Ebsdorfergrund, Germany) and centrifuged (1000 x g, 30 min, 4 °C). The interphase containing mononuclear cells (MNC) was collected and placed in a fresh 50 mL tube. This was filled up to 50 mL with PBS and centrifuged (500 x g, 10 min at 4 °C). The supernatant was discharged and the pellet resuspended. Erythrocytes lysis step was performed by adding 20 mL DW, mixing it for 3 s and then adding 20 mL 2xPBS. After centrifuging (250 x g, 10 min at 4 °C) the supernatant was discharged and the pellet was resuspended in 25 mL PBS followed by centrifugation (120 x g, 10 min at 4 °C). The final cell pellet was resuspended and adjusted to 10 × 10^6^/mL in PBS. The purity of the MNC separation was determined flow cytometrically on an SSC-A vs FSC-A density plot (S3-A).

### Monocyte subset determination

Separated MNC were placed in a 96 well round bottom plate (2 × 10^6^ MNC /well) and 30 μL of a mixture of two directly conjugated, ovine cross-reactive, monoclonal mouse anti-human antibodies was added: anti-CD14-RPE (BIO-RAD, MCA1568PE, 100 TESTS/mL, final dilution 1:10), anti-CD16-FITC (BIO-RAD, MCA5665F, 0.1 mg/mL, final dilution 1:45). The mixture was incubated for 30 min at 4 °C. Cells were washed twice with 200 μL membrane immunofluorescence (MIF) buffer (PBS + 2.5 g/L bovine serum albumin + 0.1 g/L of NaN_3_). Afterwards, 100 μL PI was added to exclude the dead cells, after gating MNC according to their FSC-A and SSC-A properties (Figure 3S-A, B, C). Three ovine monocyte subsets were defined flow cytometrically based on their CD14 and CD16 expression: classical monocytes (cM, CD14+/CD16-), intermediate monocytes (intM, CD14+/CD16+), and nonclassical monocytes (ncM, CD14−/CD16+) (Figure 3S-D). Preliminary double staining of cells with concentration-matched isotype controls (IgG2a-PE, BIO-RAD MCA929PE, IgG2a-FITC, BIO-RAD MCA929F) ensured that murine IgG2a antibodies display no unspecific reactivity with sheep monocytes. Total counts of monocyte subsets were calculated by multiplying absolute monocyte counts with monocyte subset percentages obtained after flow cytometric analysis.

### Lymphocyte proliferation capacity

Separated MNCs (10 × 10^6^/mL) were labelled with carboxyfluorescein succinimidyl ester (CFSE, 1.5 μg/mL in BPS) (C1157, ThermoFisher Scientific In., Waltham, MA, USA) and incubated for 10 min at 37 °C. The double volume of culture medium (RPMI 1640 medium, SIGMA-Aldrich®, Darmstadt, Germany) supplemented with 10% foetal calf serum (Biochrom AG, Berlin, Germany), and 100 U/mL Penicillin/Streptomycin (Invitrogen GmbH, Karlsruhe, Germany) was added and the cell suspension was centrifuged (500 x g, 10 min at 4^ο^C). The supernatant was discharged, 50 mL PBS was added and the suspension was centrifuged again (500 x g, 10 min at 4 °C). The last cell pellet was resuspended in 4 mL culture medium and adjusted to 3 × 10^6^/mL. Afterwards, the CFSE-labelled MNCs (3 × 10^5^ cells/well) were stimulated with Concanavalin A (ConA, 4 μg/mL, Sigma-Aldrich, Biochemie GmbH, Hamburg, Germany) in 96-well round bottom plates. Each setup was done in duplicate. Set ups without ConA served as controls. Plates were incubated for 4 days (37 ^ο^C, 5% CO_2_ in air). Thereafter, the plates were centrifuged (351 x g, 4 min, 8 °C), the supernatant was removed and the cells were incubated with directly labelled with a murine monoclonal antibody specific for sheep CD4 (anti sheep CD4-Alexa Fluor®-647, BIO-RAD MCA2213A647, 1:160) and a monoclonal antibody cross reactive with sheep CD8 (anti bovine CD8-RPE, BIO-RAD MCA837PE, 1:40). Preliminary staining of cells with concentration-matched isotype controls (IgG2a-PE, BIO-RAD MCA929PE, IgG2a- Alexa-Fluor-647, BIO-RAD MCA MCA929A647) ensured that murine IgG2a antibodies display no unspecific reactivity with sheep lymphocytes. After a 30 min incubation period at 4 °C cells were washed twice with MIF buffer as described above and resuspended in MIF buffer containing 2 μg/ml PI. Labelled cells were analysed flow cytometrically. CFSE fluorescence of viable (PI-negative) mononuclear cells was plotted against the cell size (FSC-A) (Figure S4-A, B). Cells displaying reduced CFSE fluorescence were identified as activated/proliferating cells. (Figure 4S-C). The proliferative capacity of T-cell subsets was determined in CFSE versus CD4-Alexa 647 and CFSE versus CD8-PE density plots, respectively (Figure S4-D, E).

### Faecal worm eggs count

Rectal faecal samples (10–15 g) were collected from individual animals at d 14 a.p, 1 p.p., 14 p.p and 30 p.p., and transported to the diagnostic laboratory of the Clinic for Swine, Small Ruminants and Forensic Medicine, University of Veterinary Medicine Hannover, Foundation, Hannover, Germany.

Faecal samples were examined using saturated Nacl as a flotation method [61]. With this method roundworms were differentiated microscopically according to the size and shape of the eggs. The roundworm species with eggs of the same shape (e.g. *Haemonchus contortus*, *Teladorsagisa circumcinta*, *Trichostrongylus colubriformis* and others) were not differentiated.

Briefly, 10 g faecal samples were suspended in D.W, sieved through a grid into a beaker in a volume of 250 ml and allowed to stand for 30 min. 2 mL of the sediment was resuspended in 9 mL saturated Nacl and centrifuged (90 x g/3 min). Three drops were taken from the surface of the liquid and placed in glass slide which covered with cover slip and examined under a microscope (10X), results were expressed as eggs per gram.

Sodium silicate solution was used for sedimentation of egg of liver fluke worms (*Fasciola spp.*). For this method 2 mL of the sediment was resuspended in 9 mL sodium silicate, 200 μL of methylene blue 3% were added and mixed well, afterwards the tubes were centrifuged (800 x g/10 min). Three drops were taken from the surface of the liquid and placed in glass slide which then was covered with cover slip and examined under the microscope (10x). The results of this investigation were negative, since eggs of liver fluke worms were not found.

### Eosinophils count

Blood was obtained by jugular vein puncture into K_2_E (EDTA) vacutainer tubes (BD Vacutainer, Belliver Industrial Estate, Plymouth, UK) in parallel with the collection of the faecal samples. The percentage of lymphocytes, neutrophils, monocytes and eosinophils were determined microscopically (counting 200 leukocytes in thin Giemsa May-Grunwald-stained blood smears). Eosinophils total counts were calculated by multiplying the percentage of eosinophils among leukocytes with the total numbers of leukocytes determined whith a hematology analyser (Celltac Alpha Nihon Kohden Europe GmbH, Rosbach vor der Höhe, Germany).

### Flow cytometric data evaluation

After acquisition, flow cytometric data were analysed with the Acurri BD™ C6 software. The gating strategies to identify cell populations, frequencies of phagocytosis-positive cells, proliferating cells, CD4+ and CD8+ positive cells among proliferating cells, and mean fluorescence intensities of phagocytosis-positive cells are described in Supplementary Figures S1 to S4.

### Statistical analysis

The data were expressed as mean ± SEM, n representing the number of animals per group. The unpaired t-test, two-way ANOVA test and Sidak multiple comparisons test (GraphPad Prism 8 Software, San Diego, CA, USA) were used for comparison between the different time points and groups. Correlations between selected variables (faecal worm egg counts and eosinophil numbers) were analyzed by Pearson’s correlation. Differences were considered statistically significant when *P* < 0.05. All data except faecal egg counts were normally distributed according to Shapiro-Wilk and Kolmogorov-Smirnov tests. Log-transformed faecal egg counts were normally distributed. Two-way ANOVA analysis of faecal egg counts was performed with antilogarithmic values calculated with the EXP function (Microsoft Excel).

## Supplementary Information

**Additional file 1: Figure S1**. Flow cytometric determination of ovine leukocyte composition. **(A)** Viable, propidium iodide-negative leukocytes after hypotonic lysis of heparinised blood were identified in a propidium iodide versus side scatter density plot. **(B)** Identification of singlets among viable leukocytes in an FSC-area versus FSC-height density plot. **(C)** Leukocytes gated on viable and single cells were plotted in FSC-A vs SSC-A density plot. Neutrophils (Neutro), lymphocytes [61], and monocytes (Mono) were identified based on their characteristic size (FSC) and complexity (SSC). Representative data from one animal.

**Additional file 2: Figure S2**. Flow cytometric determination of ovine neutrophil phagocytic activity in vitro. Heparinized blood was incubated with or without heat-killed FITC-labelled *S. aureus* (Cells:Bacteria = 1:50). **(A)** FSCA vs SSCA density plot of leukocytes after hypotonic lysis and identification of neutrophils (Neutro) based on their forward and side scatter characteristics. **(B)** Identification of viable, propidium iodide-negative neutrophils. (**C)** Identification of eosinophils in an FITC versus propidium iodide density plot. For assessing neutrophil phagocytosis in control set ups and samples with FITC-labelled *S. aureus* **(D)** in FITC versus SSC-A density plots, eosinophils were excluded from the analysis. Phagocytosis activity was defined as the percentage of green fluorescent cells among viable neutrophil (**D** lower right quadrant). The mean cellular phagocytic capacity was defined as the geometric mean green fluorescence of phagocytic-positive (green fluorescing) granulocytes. Representative data from one animal.

**Additional file 3: Figure S3** Flow cytometric determination of ovine monocyte subpopulations. **A**) Monocytes (Mono) were identified among blood mononuclear cells based on their size and complexity. **B**) Identification of viable, propidium-negative Mono and (**C**) identification of single cells among viable Mono. **D**) Correlated density plot of Mono stained with directly labelled monoclonal antibodies specific for CD16 and CD14 and identification of classical (cM, CD14++ CD16-), intermediate (intM, CD14++ CD16+) and non-classical monocytes (ncM, CD14- CD16++). Representative data from one animal.

**Additional file 4: Figure S4.** Flow cytometric determination of ovine blood mononuclear cell proliferation in vitro. Ovine mononuclear cells were obtained after density gradient separation and labelled with CFSE (1.5 μM). Cells were stimulated with Con A (4 μg/mL) and incubated for 4 days at 37 °C in vitro. Set-ups without ConA served as a control. After incubation cells were labelled with antibodies specific for CD4 (Alexa- 647) and CD8 (PE) and analysed for morphology (identification of mononuclear cells in FSCA vs SSCA density plot, A), and viability (propidium-iodide-negative cells, B). The CFSE fluorescence of viable mononuclear cells was plotted against the cell size (FSCA) (C). Cells showing no reduction in CFSE fluorescence were identified as resting cells. Small cells and cells with reduced CSFE fluorescence were identified as proliferating cells) (C). The proliferative capacity of T-cell subsets was determined in CFSE versus CD4-Alexa 647 (D) and CFSE versus CD8-PE density plots (E). (representative data from one animal).

## Data Availability

Data sets generated from this study are available upon request to the corresponding author.

## Abbreviations

CD: Cluster of differentiation
CFSE: carboxyfluorescein succinimidyl ester
cM: Classical monocyte
ConA: Concanavalin A
FSC-A: Forward scatter area
GMB: German black headed mutton
intM: Intermediate monocyte
MAGT1: Magnesium transporter 1
MAP: *Mycobacterium avium* paratuberculosis
Mg: Magnesium
MIF: Membrane immunofluorescence
MNC: Mononuclear cells
ncM: Nonclassical monocyte
PI: Propidium iodide
PPR: Periparturient rise
SSC-A: Side scatter area
TP: Transition period
TRPM7: Transient receptor potential melastatin family member 7
