# Supplementary material for: Blood leukocyte composition and function in periparturient ewes kept on different dietary magnesium supply
Source: BMC Vet Res. 2020 Dec 14;16:484. doi: 10.1186/s12917-020-02705-9 (PMC7734835; doi:10.1186/s12917-020-02705-9)
Supplement: Supplementary file 1 — Additional file 1: Figure S1. Flow cytometric determination of ovine leukocyte composition. (A) Viable, propidium iodide-negative leukocytes after hypotonic lysis of heparinised blood were identified in a propidium iodide versus side scatter density plot. (B) Identification of singlets among viable leukocytes in an FSC-area versus FSC-height density plot. (C) Leukocytes gated on viable and single cells were plotted in FSC-A vs SSC-A density plot. Neutrophils (Neutro), lymphocytes [61], and monocytes (Mono) were identified based on their characteristic size (FSC) and complexity (SSC). Representative data from one animal. [file 12917_2020_2705_MOESM1_ESM.pptx]

## Slide 1
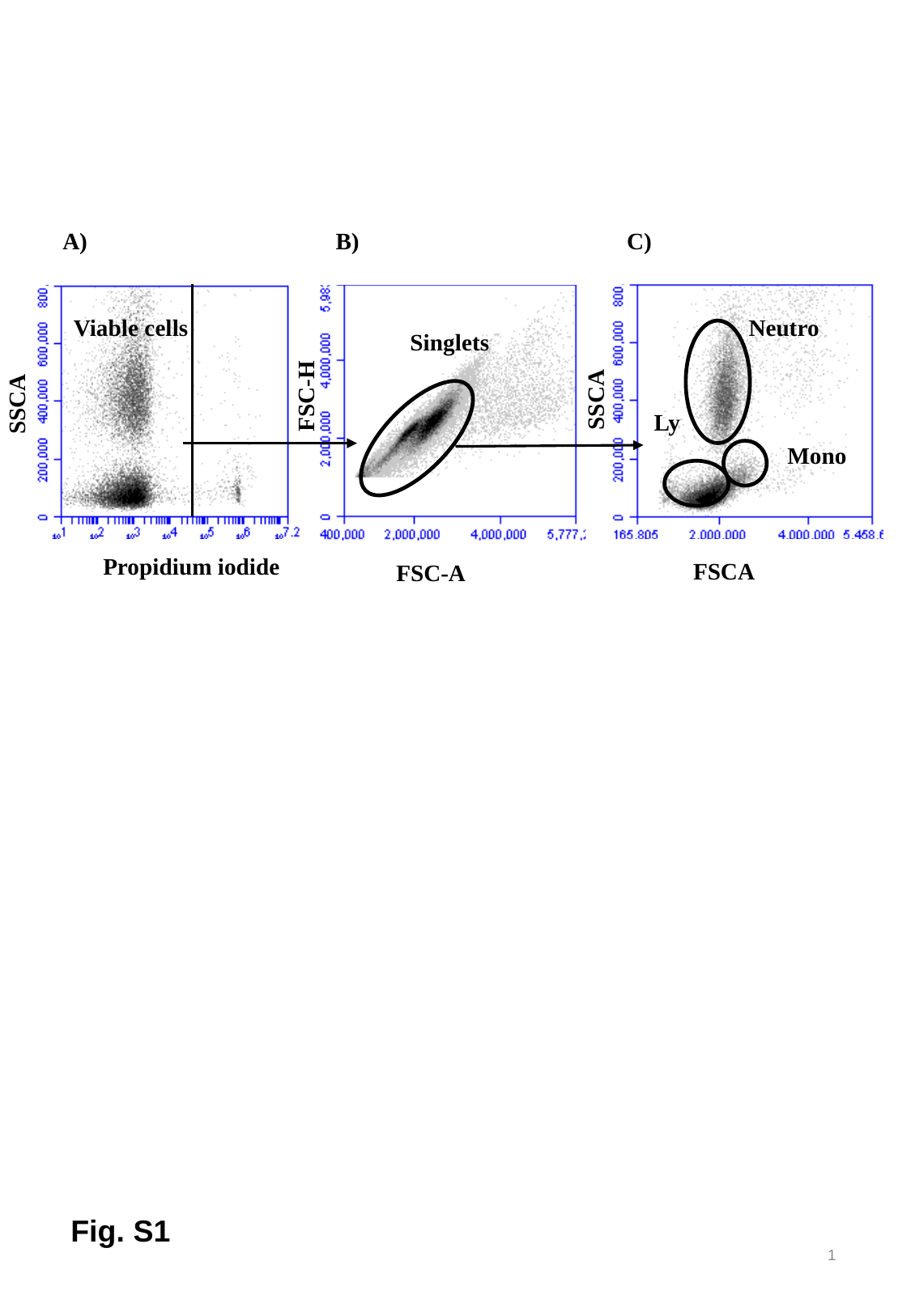

B
C
A
FSC-A
A)
B)
C)
Neutro
SSCA
SSCA
Ly
Mono
FSCA
Singlets
FSC-H
FSC-A
Viable cells
FSC-H
SSCA
SSCA
Propidium iodide
Fig. S1
1
