# Supplementary material for: Blood leukocyte composition and function in periparturient ewes kept on different dietary magnesium supply
Source: BMC Vet Res. 2020 Dec 14;16:484. doi: 10.1186/s12917-020-02705-9 (PMC7734835; doi:10.1186/s12917-020-02705-9)
Supplement: Supplementary file 3 — Additional file 3: Figure S3 Flow cytometric determination of ovine monocyte subpopulations. A) Monocytes (Mono) were identified among blood mononuclear cells based on their size and complexity. B) Identification of viable, propidium-negative Mono and (C) identification of single cells among viable Mono. D) Correlated density plot of Mono stained with directly labelled monoclonal antibodies specific for CD16 and CD14 and identification of classical (cM, CD14++ CD16-), intermediate (intM, CD14++ CD16+) and non-classical monocytes (ncM, CD14- CD16++). Representative data from one animal. [file 12917_2020_2705_MOESM3_ESM.pptx]

## Slide 1
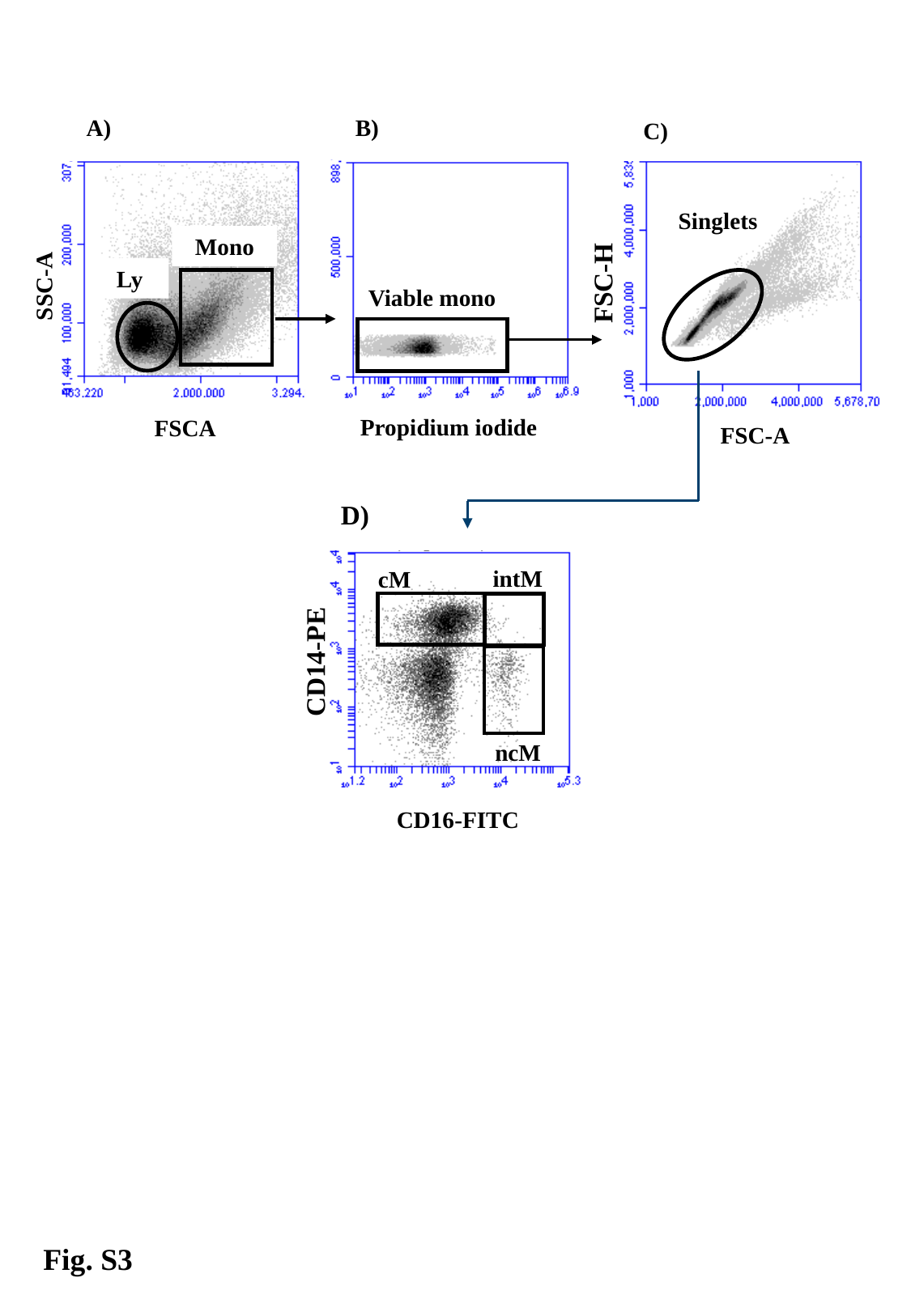

A)
B)
C)
Singlets
Mono
FSC-H
Ly
SSC-A
Viable mono
Propidium iodide
FSCA
FSC-A
D)
intM
cM
CD14-PE
ncM
CD16-FITC
Fig. S3
