# Supplementary material for: Blood leukocyte composition and function in periparturient ewes kept on different dietary magnesium supply
Source: BMC Vet Res. 2020 Dec 14;16:484. doi: 10.1186/s12917-020-02705-9 (PMC7734835; doi:10.1186/s12917-020-02705-9)
Supplement: Supplementary file 4 — Additional file 4: Figure S4. Flow cytometric determination of ovine blood mononuclear cell proliferation in vitro. Ovine mononuclear cells were obtained after density gradient separation and labelled with CFSE (1.5 μM). Cells were stimulated with Con A (4 μg/mL) and incubated for 4 days at 37 °C in vitro. Set-ups without ConA served as a control. After incubation cells were labelled with antibodies specific for CD4 (Alexa- 647) and CD8 (PE) and analysed for morphology (identification of mononuclear cells in FSCA vs SSCA density plot, A), and viability (propidium-iodide-negative cells, B). The CFSE fluorescence of viable mononuclear cells was plotted against the cell size (FSCA) (C). Cells showing no reduction in CFSE fluorescence were identified as resting cells. Small cells and cells with reduced CSFE fluorescence were identified as proliferating cells) (C). The proliferative capacity of T-cell subsets was determined in CFSE versus CD4-Alexa 647 (D) and CFSE versus CD8-PE density plots (E). (representative data from one animal). [file 12917_2020_2705_MOESM4_ESM.pptx]

## Slide 1
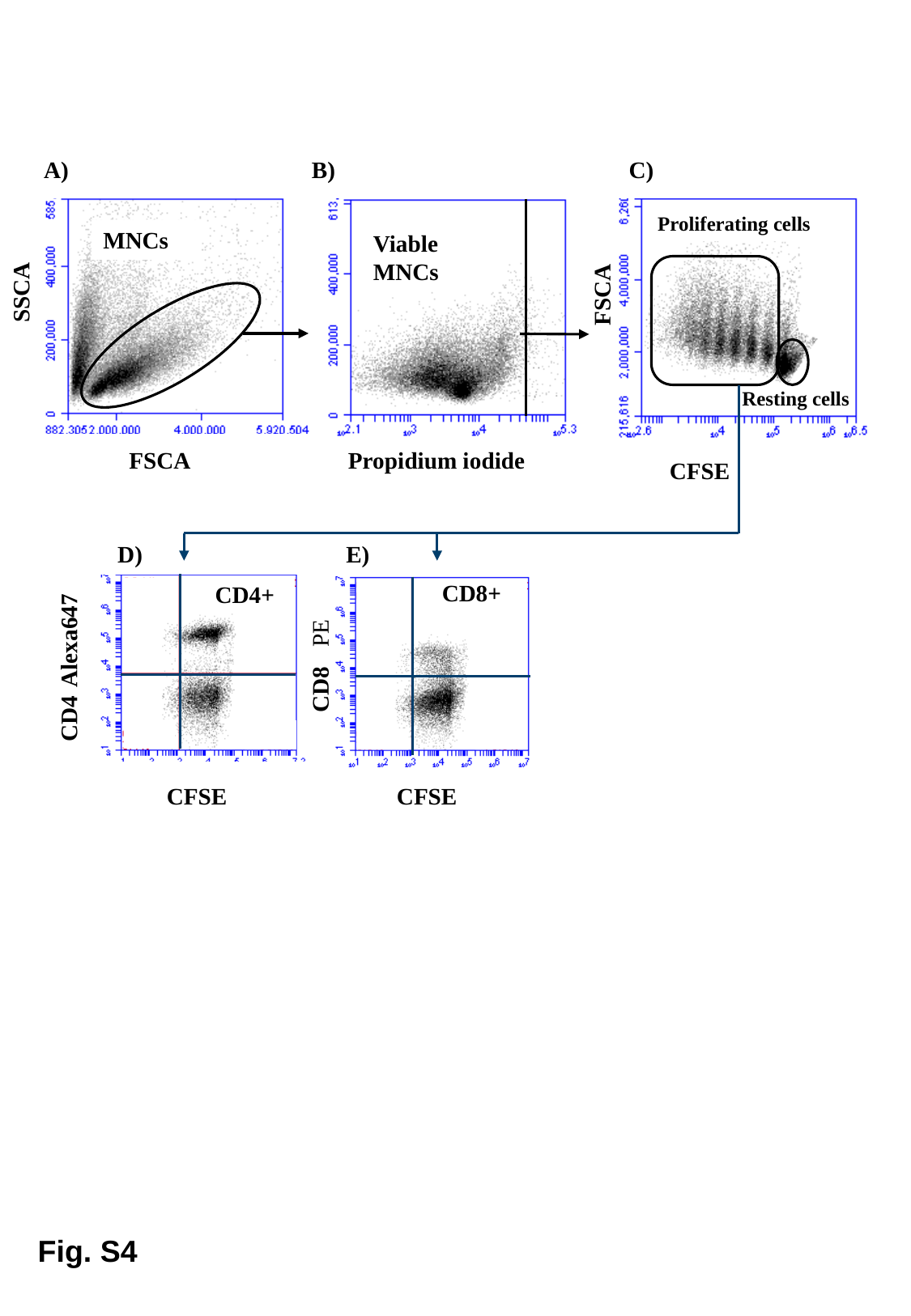

A)
B)
C)
Proliferating cells
MNCs
Viable MNCs
FSCA
SSCA
FSCA
Propidium iodide
CFSE
D)
E)
CD4+
CD8+
CD4-Alexa647
CD8 - PE
CFSE
CFSE
Resting cells
Fig. S4
